# Short‐ and long‐term antidepressant effects of ketamine in a rat chronic unpredictable stress model

**DOI:** 10.1002/brb3.749

**Published:** 2017-06-23

**Authors:** Yinghong Jiang, Yiqiang Wang, Xiaoran Sun, Bo Lian, Hongwei Sun, Gang Wang, Zhongde Du, Qi Li, Lin Sun

**Affiliations:** ^1^ Department of Psychology Weifang Medical University Shandong China; ^2^ School of Bioscience and Technology Weifang Medical University Shandong China; ^3^ Laboratory for Cognitive Neuroscience Weifang Medical University Shandong China; ^4^ Department of Neurology Chinese People's Liberation Army eighty‐nine Hospital Shandong China; ^5^ Department of Psychiatry and Centre for Reproduction Growth and Development University of Hong Kong HongKong China

**Keywords:** antidepressant, behavioral test, chronic unpredictable stress, depression, ketamine

## Abstract

**Objective:**

This research was aimed to evaluate the behaviors of short‐ or long‐term antidepressant effects of ketamine in rats exposed to chronic unpredictable stress (CUS).

**Background:**

Ketamine, a glutamate noncompetitive NMDA receptor antagonist, regulates excitatory amino acid functions, such as anxiety disorders and major depression, and plays an important role in synaptic plasticity and learning and memory.

**Methods:**

After 42 days of CUS model, male rats received either a single injection of ketamine (10 mg/kg; day 43) or 15 daily injections (days 43–75). The influence of ketamine on behavioral reactivity was assessed 24 hr (short‐term) or 7 weeks after ketamine treatment (long‐term). Behavioral tests used to assess the effects of these treatments included the sucrose preference (SP), open field (OF), elevated plus maze (EPM), forced swimming (FS), and water maze (WM) to detect anxiety‐like behavior (OF and EPM), forced swimming (FS), and water maze (WM).

Results: Short‐term ketamine administration resulted in increases of body weight gain, higher sensitivity to sucrose, augmented locomotor activity in the OF, more entries into the open arms of the EPM, along increased activity in the FS test; all responses indicative of reductions in depression/despair in anxiety‐eliciting situations. No significant differences in these behaviors were obtained under conditions of long‐term ketamine administration (*p* > .05). The CUS + Ketamine group showed significantly increased activity as compared with the CUS + Vehicle group for analysis of the long‐term effects of ketamine (*p < .05). Nor were significant differences obtained in learning and memory performance in rats receiving ketamine (*p* > .05).

**Conclusion:**

Taken together these findings demonstrate that a short‐term administration of ketamine induced rapid antidepressant‐like effects in adult male rats exposed to CUS conditions, effects that were not observed in response to the long‐term treatment regime.

## 1 INTRODUCTION

Depression is the most common mental disorder in community settings and a major cause of disability worldwide. It includes a wide range of mental health problems manifested by the absence of a positive affect (a loss of interest and enjoyment in ordinary events and experiences), despondent mood and a number of associated emotional, cognitive, physical, and behavioral symptoms (Health, 2010; Krishnan & Nestler, 2008). It has been reported that the prevalence of depression in most countries ranges from 8 to 12%, which results in enormous personal suffering, as well as substantial social and economic burdens (Kessler et al., 2003; Pincus & Pettit, 2001). The world health organization estimates that by 2020 depression is projected to become the second most common cause of disability‐adjusted life years worldwide (Murray & Lopez, 1997).

Normally, antidepressants such as selective serotonin reuptake inhibitors (SSRIs) and selective norepinephrine inhibitors (SNRIs) are used in the treatment of this disorder (Ahuja, 2008). It is generally believed that the increase in neuronal monoamine neurotransmitter transport that occurs in response to these agents can improve the adaptability of neurons in the limbic brain regions that control mood and depression, to achieve their therapeutic effect (Warner‐Schmidt et al., 2010). However, these typically prescribed antidepressants have significant limitations, including low effective rates upon initial use (effective rates to the first treatment account for only one‐third, and effective rates with multiple trials accounts for only up to two‐thirds of treated patients), substantial latencies for a therapeutic response (weeks to months) and serious side effect (such as decreased libido and weight increases) (Trivedi et al., 2006). Such shortcomings are particularly problematic for an illness that is associated with high rates of suicide, further highlighting a major unmet need for the development of novel, rapid‐acting, and more efficacious antidepressant agents.

Several lines of evidence suggest that dysfunction of the glutamate neurotransmitter system is also associated with the pathophysiology of mood disorders, such as major depressive disorder (MDD) and bipolar disorder (BP) (Hashimoto, 2009, 2011; Sanacora, Zarate, Krystal, & Manji, 2008; Tokita, Yamaji, & Hashimoto, 2012; Zarate et al., 2010). Results from postmortem studies reveal that glutamate levels are reduced in the prefrontal cortex (PFC) in MDD, which indicates that glutamate neurotransmission in MDD patient shows abnormal functioning with regard to both pathophysiology and glutamate receptor expression (Zhao et al., 2012). Ketamine, a noncompetitive glutamate NMDA receptor antagonist, regulates excitatory amino acid function and plays an important role in synaptic plasticity, learning, and memory. It has been reported that subanesthetic doses of ketamine (10 mg/kg) reduce depression symptoms in rats subjected to the depression model of learned helplessness (Beurel, Song, & Jope, 2011). Moreover, double‐blind, placebo‐controlled randomized clinical trials have also demonstrated the efficacy of a single dose of ketamine in treating patients with refractory MDD and BP (Berman et al., 2000; Diazgranados et al., 2010; Zarate et al., 2006). The rapid onset of ketamine action makes it a highly attractive drug for patients with mood disorders, although the mechanistic action is unknown (Mathews, Henter, & Zarate, 2012). Discovery of the rapid antidepressant actions of ketamine, which acts by a mechanism completely different from typical monoamine reuptake inhibitors, represents a major advance in the field of depression. However, as the widespread use of ketamine is limited by the potential for toxicity and abuse, studies are being conducted in animal models to elucidate the mechanisms underlying the actions of ketamine as an approach to develop safe, rapidly acting agents.

In an attempt to further understand some of the parameters of ketamine's effects as an antidepressant, the purpose of this study was to investigate the effects of ketamine in the rat model of chronic unpredictable mild stress (CUS). This model is widely used as a preclinical animal model of depression (Cryan & Holmes, 2005; Nestler et al., 2002). The specific goals of this study were to examine whether ketamine could improve depression‐like behaviors seen in rats subjected to CUS, and to determine the rapidity and duration of these antidepressant effects of ketamine.

## 2 MATERIALS AND METHODS

### 2.1 Animals

Male Sprague–Dawley (SD) rats (250–350 g) obtained from the animal center at WeiFang Medical University were used for this study. The age of the rats at the start of experiment was day 35–49, which roughly approximates adolescence in humans (Andersen & Navalta, 2004; Spear, 2000). Procedures involving animal use were in accordance with the National Institutes of Health Guidelines (Use of Laboratory Animals) and were approved by the WeiFang Medical University Animal Care and Use Committees. The rats were allowed a 1‐week period of acclimatization to their housing conditions before commencing stress exposure. During the acclimatization period food and water were provided adlibitum. Rats were housed in temperature (23 ± 2°C) and humidity (50 ± 5%) controlled rooms and maintained on a 12 hr/12 hr light/dark cycle.

### 2.2 Chronic unpredictable stress paradigm

To establish the chronic mild unpredictable stress animal model, 10 different stressors, with two stressors per day were administered. The stressors used were as reported previously (Li et al., 2011; Spear, 2000) and included: lights off for 3 hr (10 am to 1 pm), lights on overnight, strobe light overnight, 45° tilted cages, food and water deprivation, crowded housing, isolation housing, tail clip, wet cage, and noise. The stressors were administered over a 42‐day period. Throughout the duration of the experiment, all rats remained healthy with no obvious indications of sickness and no deaths.

### 2.3 Drug specification and experimental design

After establishing the CUS model, rats received ketamine (10 mg/kg, ip.) once daily for either 1 day or 15 consecutive days. The influence of ketamine on behavioral reactivity was then assessed at either 1 day (short‐term) or at 7 weeks (long‐term) after the onset of the first ketamine administration. This subanesthetic dose of ketamine was chosen as it is regarded to represent a recreational dose for use in rodents with a LD50 of 600 mg/kg at 4 hr and was consistent with dosages reported in the literature (Enomoto & Floresco, 2009; Quirk, Sosulski, Feierstein, Uchida, & Mainen, 2009; Sun et al., 2011). A placebo group received injections of physiological saline (0.9% sodium chloride, ip), and an untreated (normal) control group received no treatments during the 15 days of ketamine/saline injection. So 28 rats were randomly assigned into three groups as follows: “CUS + Ketamine,” “CUS + Vehicle,” and “Control” group.

### 2.4 Behavioral tests

**Figure 1:**
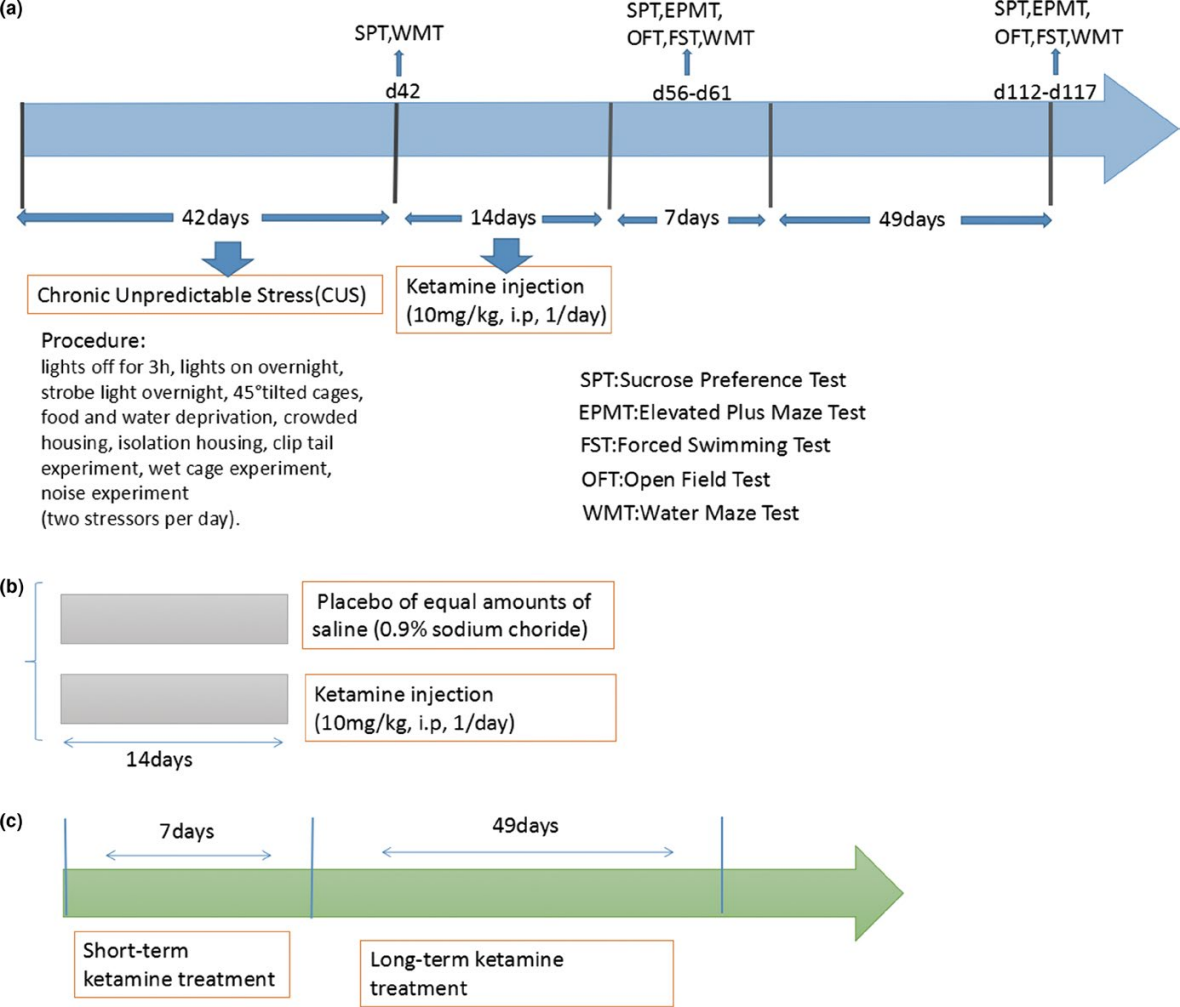
(a) Flow diagram of experiment. A total of three groups of behavior experiments included SPT, WMT, EPMT, OFT, and FST. (b)The experiment included ketamine‐treated (katemine injection 10 mg/kg, 1/day, i.p. *N* = 10) group, CUS model (placebo of equal amounts of saline. *N* = 10) group and control (without any further processing. N = 8) group or simple  “CUS + Ketamine,” “CUS + Vehicle,” and “Control” for short. (c) The study of the antidepressant short‐term and long‐term efficacy of ketamine in rats using the behavioral experiments

Rats were subjected to two behavioral assays on the final day of ketamine or saline administration to verify the presence of stress in this CUS model, the Sucrose Preference Test (SPT) and the Water Maze Test (WMT) (see Figure 1 for experimental groups/testing sequence). These behavioral tests were conducted at 4 hours after injection, as the metabolites of ketamine are cleared by urinary excretion by 4 hours after injection (Sun et al., 2011). The influence of ketamine on the five behavioral tests described below was assessed at 24 hr (short‐term) and at 7 weeks (long‐term) after ketamine treatment. Behavioral assessments were recorded using a camera (SMART, Panlab SL, and Barcelona, Spain) and were scored by two raters who were blind as to the treatment condition of the groups. No statistically significant differences were obtained between scores of the raters (inter‐rater reliability = 0.91). All tests were performed between 800 and 1700 hr.

### 2.5 Sucrose preference test

Sucrose preference is considered to provide an index of anhedonia (Shi et al., 2012; Warner‐Schmidt & Duman, 2008). The rats were trained to adapt to a 1% sucrose solution (w/v) for 48 hr at the beginning of the experiment. For this two‐bottle choice test (Bolanos, Barrot, Berton, Wallace‐Black, & Nestler, 2003), one bottle was filled with 1% sucrose solution and the other filled with water and both were then placed in the cage as described previously (Suo et al., 2013). The positions of water and sucrose bottles were altered during the training sessions. For the SPT, rats were deprived of water for 21 hr and placed individually in a cage containing two identical bottles for a 3‐hr period. The amount of sucrose and water consumed (mL) was recorded. A sucrose preference percent (%) was calculated by measuring sugar water intake divided by the sum of sugar water intake and water intake.

### 2.6 Elevated plus maze test

The elevated plus maze (EPM) is based on a rat's natural fear of open, unprotected and elevated spaces (Suo et al., 2013). Each rat was first placed in the central zone of the EPM with its head oriented to the closed arm. The rat was allowed to freely explore the maze for 5 min as described previously (Franceschelli, Sens, Herchick, Thelen, & Pitychoutis, 2015; Suo et al., 2013) and their behavior was recorded using a video camera placed above the EPM. The EPM was cleaned with 70% ethanol between tests to prevent interference resulting from any residual odors of the previous rat. The number of entries into and time spent in the open and closed arms were recorded. A retention time (time spent in the open arm) and the ratio number of entries into the open arm were calculated by Smart electronic equipment.

### 2.7 Open field test

On days 56 and 113 of the experiment (15 and 57 days after their final ketamine injection), selected rats from each group were evaluated in the open field test during the dark cycle of their photoperiod (1300‐1700 hr) (Walsh & Cummins, 1976). This apparatus, which has been described previously (Ma et al., 2013, 2011), consisted of a square box with dimensions, 80 × 80 × 50 cm, and the field was divided into 25 squares with computer virtual grid lines for analysis using the Smart3.0 software. The rat's behavior was recorded within 5 min. For each trial, rats were allowed to activity for 5 min. At the start of the test, the rat was placed in the corner of the open field. The numbers of grids crossed (all four limbs), up‐right postures and carding, fecal grains and entrances into the central zone of the apparatus were all recorded in this test. The apparatus was cleaned with 70% ethanol between each test to eliminate any residual olfactory cues of the previously tested rat.

### 2.8 Forced swimming test

The FST was performed using methods previously described by Sadeghi, Peeri, & Hosseini, (2016). The equipment for this test consisted of a glass barrel (height = 46 cm, diameter = 20 cm) filled with 36 cm of water and maintained at 23 ± 1°C. Prior to testing, the rats were placed in the water for a 15‐min period to adapt to the apparatus**.** Twenty‐four hours later, they were again placed in the cylinders for the 5 min FST. The latency of immobility, as defined by the time at which the rat was no longer engaged in actively swimming, was then recorded. After testing, rats were removed, placed in a normal heat preservation breeding cage with padding, and covered with an absorbent towel.

### 2.9 Water maze test

The WMT is used to measure spatial navigation learning and memory in rats.(Sterneck et al., 1998). All groups of rats were trained for 3 days, and the WMT was performed as described previously (Sun et al., 2011). A black circular tank (150 cm in diameter) was filled to a depth of 25 cm with water and maintained at 23 ± 1°C. For each trial, rats were allowed to search for the hidden platform for a 90 s period. The platform was hidden 1 cm below the water surface in the center of one quadrant of the pool. If a rat did not locate the platform after 90 s, it would be guided to the platform and allowed to remain on the platform for 20 s to recognize the location. The rats received three such consecutive trials each day with an intertrial interval of 30 s. Four prominent signs (square, heart‐shape, moon and triangle) were placed around the tank at four fixed points, with one in each quadrant. The water was changed each day's experiment. The escape latency time for the rat to locate and climb onto the platform was recorded.

### 2.10 Statistical analyses

Statistical analysis was performed using SPSS 18.0 and Graphpad Prism 5. All data were expressed as mean ± standard deviation (SD) or median (10th to 90th percentile) for the groups. Behavioral data were analyzed using a mixed‐design analyses of variance (ANOVAS) followed by Fisher least significant difference (LSD) post hoc test. Results were considered statistically significant when *p* < .05.

## 3 RESULTS

### 3.1 Effects of ketamine on body weight gain

**Figure 2:**
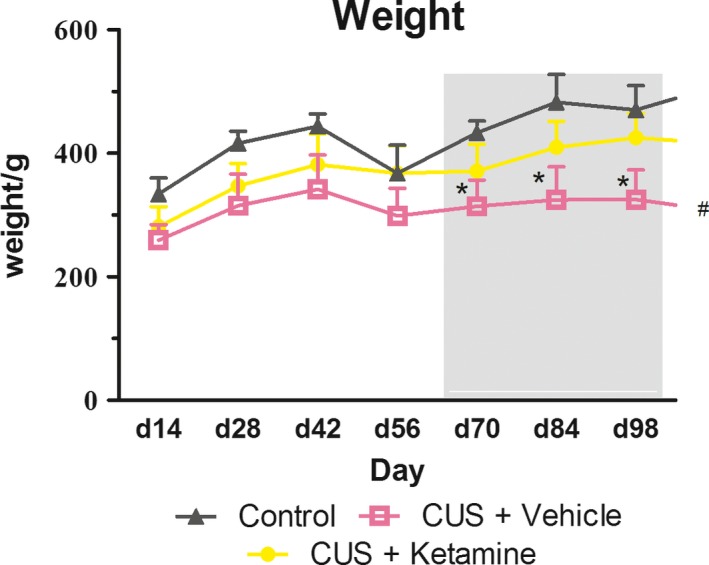
The final body weights were expressed as the percentage of the body weights at 7‐day. As shown in the figure, the body weight tendency of “CUS + Ketamine (*N* = 10),” “CUS + Vehicle (*N* = 10),” and “Control (*N* = 8)” group rates were expressed in three different shapes of line, respectively. Figure in the gray area from long‐term efficacy of ketamine (experiment start days 63–112). Data are represented as body weight in grams and results were group means +standard deviations. *p < .05 were expressed between CUS + Ketamine and CUS + Vehicle group statistically significant. ^#^ *p *< .05 were expressed between Control and CUS + Vehicle group statistically significant

Reductions in body weight can be used as an index of physiological responses indicative of chronic stress. In our experiment, CUS + Vehicle rats gained weight at a lower rate than the control group, with the result being that body weights of rats in the CUS + Vehicle group on day 42 were significantly decreased (*p < .001; Figure 2). While rats treated with ketamine showed significantly reduced weight gains as compared with normal controls (^#^ *p *< .05), their weight gains were significantly increased as compared with CUS + Vehicle rats not receiving ketamine (^#^ *p *< .05) throughout the duration of the experiment. The differences between these two latter groups were enhanced as a function of the duration of the experiment – day 70 (*p < .001), day 84 (*p < .002), and day 98 (*p < .001).

### 3.2 Effects of ketamine's on reward‐related behavior

**Figure 3:**
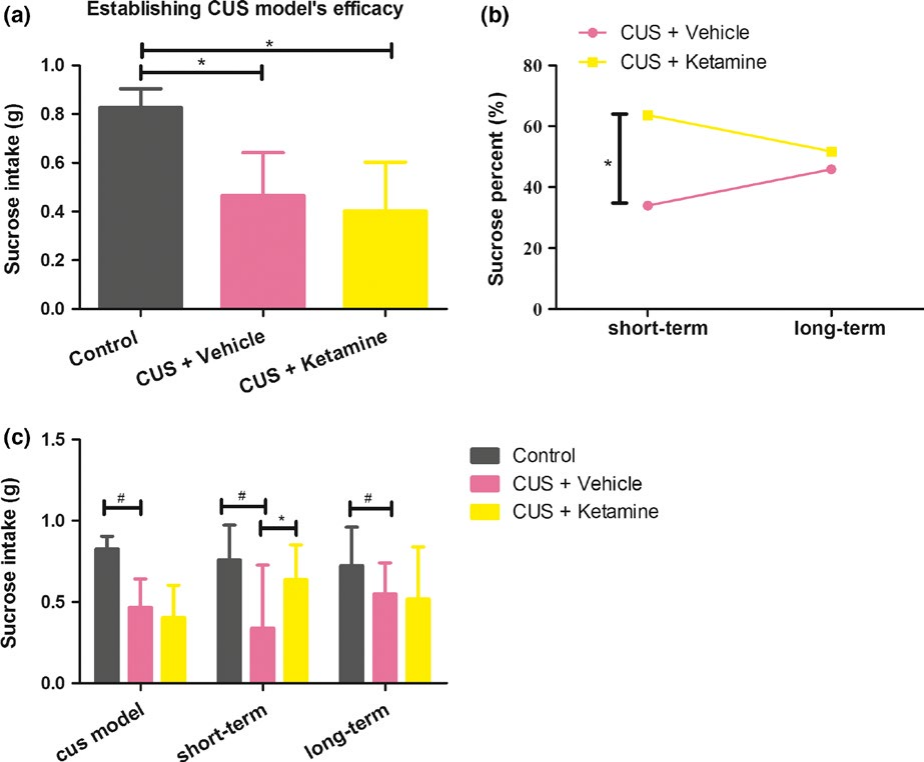
(a) The differences sucrose intake of rats exposed to establishing CUS model efficacy. *p < .05 were expressed statistically significant, “CUS + Ketamine (*N* = 10),” “CUS + Vehicle (*N* = 10),” and “Control (*N* = 8).” (b) The rapid and long‐term effects of ketamine in this CUS model. (c) The sucrose intake of rats in the study. *p < .05 were expressed between CUS + Ketamine and CUS + Vehicle group statistically significant. ^#^ *p *< .05 were expressed between Control and CUS + Vehicle group statistically significant. And the results were group means + standard deviations

The sucrose preference test provides an index of stress‐induced anhedonia (Figure 3c). Exposure of rats to the 42‐day stress regime was successful in eliciting an anhedonia‐like condition as sucrose consumption in stressed rats was significantly lower than that of the untreated control group (*F* (2, 27) = 16.572, *p < .001: CUS + Ketamine group versus Control group, *p < .001 and CUS + Vehicle group versus Control group, *p < .001; Figure 3a). In the short‐term, ketamine treatment significantly increases the percent of sucrose intake as compared with the CUS + Vehicle group (*p < .036). Although sucrose intake of the ketamine group was less than that of the CUS + Vehicle group, no statistically significant was found**.**

### 3.3 Effects of ketamine on anxiety‐like behavior

The OF and EPM tests were used to assess anxiety‐like behavior in rats. In the OF, reductions in carding and crossing numbers indicate higher levels of anxiety/depression. The results of the OF test in response to the short‐term ketamine treatment indicated that carding and fecal grains in CUS + Ketamine group were significantly increased as compared to CUS + Vehicle group**,** but the scores for crossing and up‐right posture were not statistically significant (carding: *F*(2, 27) = 4.28, *p < .025;fecal grains: *F*(2, 27) = 3.89, *p < .034). Analysis of results for long‐term ketamine administration indicated no overall statistically significant differences among the three groups in the OF test (Tables 1, 2).

**Table 1:** CUS + Ketamine group significantly reduced number in up‐right, carding and fecal grains compared with other groups during short‐time effects. Values are means±standard deviations

	Control	CUS + Vehicle	CUS + Ketamine	*F*	*p*
Crossing	50.30 ± 16.30	34.50 ± 19.49	37.50 ± 16.68	2.26	.125
Up‐right	17.90 ± 6.92	15.13 ± 4.85	14.60 ± 7.03	0.74	.487
Carding	6.10 ± 2.88	2.88 ± 1.89	3.20 ± 2.90	4.28	.025^a^
Fecal grains	3.80 ± 3.82	0.01 ± 0.01	4.50 ± 4.62	3.89	.034^a^

a *p *< .05 exposed to repeated ANOVA was significantly different.

**Table 2:** CUS + Ketamine group significantly reduced number in up‐right, carding and fecal grains compared with other groups during short‐time effects

	Control	CUS + Vehicle	CUS + Ketamine	*F*	*p*
Crossing	44.70 ± 16.99	36.88 ± 27.72	39.44 ± 26.95	0.25	.778
Up‐right	16.00 ± 6.50	13.75 ± 8.80	14.00 ± 8.05	0.24	.790
Carding	3.40 ± 2.50	2.62 ± 2.26	2.33 ± 1.41	0.63	.535
Fecal grains	2.60 ± 2.84	1.25 ± 2.38	2.22 ± 3.07	0.54	.591

Values are means ± standard deviations.

**Figure 4:**
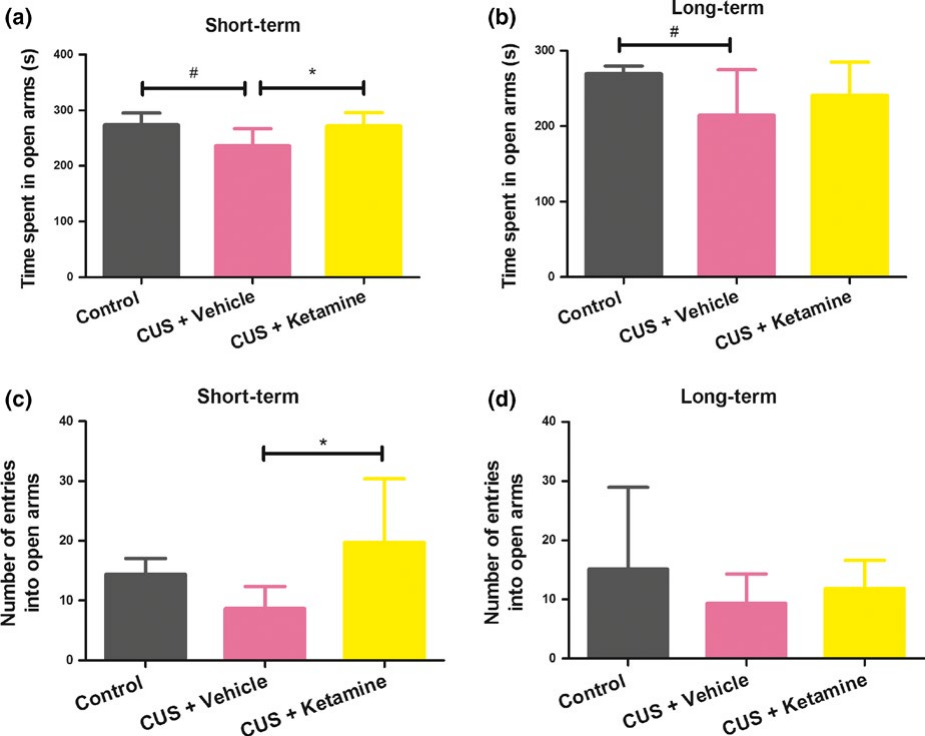
Effects of 1 and 15 days of ketamine exposure (10 mg/kg; once daily) on anxiety‐like behavior. The results were group means + standard deviations, “CUS + Ketamine (*N* = 8),” “CUS + Vehicle (*N* = 10),”and “Control (*N* = 8).” (a, c) Rats short‐term effects of repeated ketamine exposure on anxiety‐like behavior. (b, d) Rats long‐term effects of repeated ketamine exposure on anxiety‐like behavior. *p < .05 were expressed between CUS + Vehicle and CUS + Vehicle group statistically significant. ^#^ *p *< .05 were expressed between Control and CUS + Vehicle group statistically significant

For the EPM, the CUS + Ketamine group showed significantly higher ratios of entries into the open arm (*F*(2, 23) = 6.530, *p < .006) and longer durations within the open arms (*F*(2, 23) = 6.250, *p < .007) as compared with the CUS + Vehicle group in the short‐term condition (Figure 4a, c). However, results between the CUS + Ketamine and CUS + Vehicle groups were not significantly different on day 115 (Figure 4b, d). CUS + Vehicle rats receiving, short‐ or long‐term ketamine treatment spent significantly less time in the open arms of the EPM as compared with the Control group (short‐term: *p < .005; long‐term: *p < .017). The CUS + Ketamine group showed no significantly of entries into the open arm. (Figure 4b, d).

### 3.4 Effects of ketamine on behavioral despair

**Figure 5:**
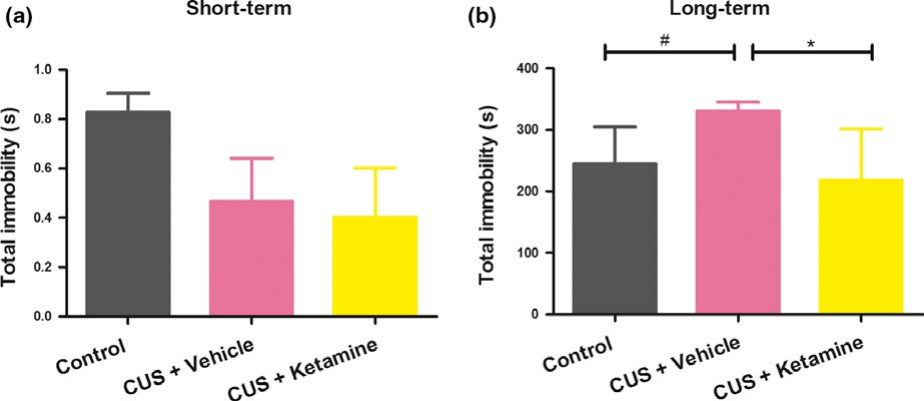
(a,b) Short‐ and long‐term of total immobility time showed different results. The results were group means + standard deviations, “CUS + Ketamine (*N* = 8),” “CUS + Vehicle (*N* = 10),”and “Control (N = 8).” *p<.05 were expressed between CUS + Vehicle and CUS + Ketamine group statistically significant. ^#^ *p *< .05 were expressed between Control and CUS +Vehicle group statistically significant

Ketamine affects responses observed in the FS test, with decreases in immobility (increased activity) being indicative of reductions in despair. While analysis of the short‐term effects of ketamine in the FS test indicated that these CUS + Ketamine rats were more active (decreased immobility) as compared to the other two groups, but the results were not statistically significant. For analysis of the long‐term effects of ketamine, the CUS + Ketamine group showed significantly increased activity as compared with the CUS + Vehicle group (*p < .001), and the CUS + Vehicle group also decreased activity as with the Control group (*p < .005) (*F*(2, 23) = 9.261, *p < .001; Figure 5b).

### 3.5 Effects of ketamine on learning and memory

**Figure 6:**
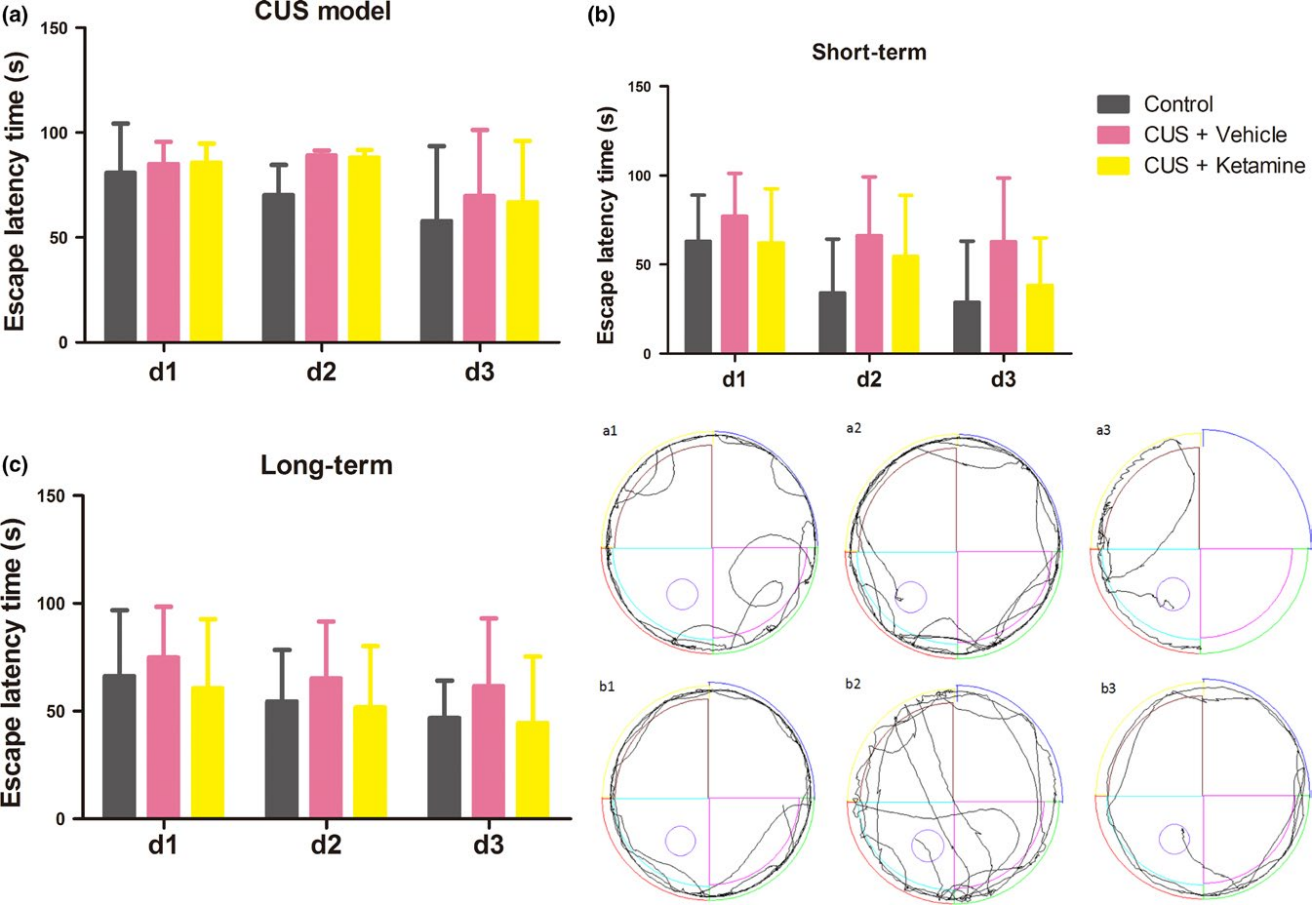
Water maze test results of different rats groups. After training for 3 days, water maze test was performed 4 hr after the last ketamine or saline injection to rats. (a) The differences escape latency time of rats exposed to establishing CUS model efficacy during 3 days. (b,c) Short‐ and long‐term functional consequences of ketamine of escape latency time showed different results. Repeated ANOVA analysis with LSD multiple comparison of the escape latency time were not significantly among all groups, and “CUS + Ketamine (*N* = 10),” “CUS + Vehicle (*N* = 10),” and “Control (*N* = 8).” (a1‐3) The effects of ketamine‐injected were presence of short‐term swimming trajectory. (b1‐3) The effects of ketamine‐injected were presence of long‐term swimming trajectory

As memory can also be affected with depression, we assessed memory function in these rats with use of the Water Maze Test. When tested in the water maze test, reductions in escape latencies and distance traveled to the platform provide an index of memory function. The escape latency time(s) for the rats to locate and climb onto the platform showed no statistically significant differences, although the CUS + Vehicle group rats spent more time to find the platform. Further analysis (LSD) showed that escape latencies of the CUS + Ketamine groups were increased in comparison to CUS + Vehicle group in the last day (CUS + Ketamine: 61.58 ± 23.51, CUS + Vehicle: 44.48 ± 21.17), but the result was not statistically significant. Moreover, the distance traveled indicated the differences in space exploration between short‐ and long‐term administration of ketamine (Figure 6a1‐3, b1‐3).

## 4 DISCUSSION

Several lines of evidence have suggested that ketamine rapidly enhances the structure and function of cortical synapses known to play a role in mood. It has been proposed that the antidepressant effects of ketamine involve a rapid activation of the mammalian rapamycin pathway, including increases in extracellular signals regulating kinase, protein kinase B, and brain‐derived neurotrophic factor in the hippocampus and producing increases in the number of new spines in the prefrontal cortex (Autry et al., 2011). Importantly, age of the subject and ketamine dose have proved to be significant factors in producing these antidepression effects. Subanesthetic doses of ketamine are thought to enhance glutamatergic signaling and dopamine release in the prefrontal cortex by reducing excitatory input upon gamma amino butyric acid neurons, subsequently leading to hyperactivity of corticolimbic pathways (Li et al., 2010; Moghaddam, Adams, Verma, & Daly, 1997). As adolescents have higher concentrations of NMDA receptors and typically metabolize drugs more rapidly than adults (Hein, 1987), it seems possible that the subanesthetic doses of ketamine used in the adolescent rats of our experiment involved similar mechanisms to produce the increased levels of exploratory behavior observed.

Significant differences in weight gains were obtained between CUS + Vehicle exposed rats and the Control group (Figure 6). Stressful situations are known to influence feeding behavior as it has been reported that chronic exposure to stressors alters body weights in rats (Bekris, Antoniou, Daskas, & Papadopoulou‐Daifoti, 2005; Dess, Raizer, Chapman, & Garcia, 1988). During CUS model, the hypothalamus‐pituitary‐adrenal (HPA) axis and glucocorticoids (GC) remain elevated, and the main adrenal cortical hormone in rats is corticosterone (mineralocorticoid) (Cryan, Mombereau, & Vassout, 2005). Cortisone injections enhance the body's sensitivity to insulin sensitivity to reduce food intake, leading to the weight loss (Nilsson et al., 2002). This result was consistent with our report indicating that CUS induced growth retardation, and is supported by related findings which show reduced body weights and caloric intakes in depressed animals and humans (Iniguez, Warren, & Bolanos‐Guzman, 2010; Simansky & Eberle‐Wang, 1993). In our study, the ketamine‐treated group showed significantly elevated body weights as compared with the CUS + Vehicle group throughout days 42 to112 of the experiment. From days 84 to 112, the decline in body weights within the CUS + Vehicle group was particularly significant. As described above, the central nervous system plays an important role in the control of appetite and maintenance of body weight (Coppari, Ramadori, & Elmquist, 2009). Accordingly, the effects of ketamine observed within our study likely reflect the effects exerted upon hormonal and neuronal signals regulating appetite and body weight control in young animals (Sun et al., 2011). The rapid recovery of body weight in stressed rats treated with ketamine suggests that NMDA receptors are involved in modulating feeding behavior during stressful situations. Interestingly, in adult humans and rats, chronic ketamine exposure disrupts appetite and weight gain (Cvrcek, 2008; Parise et al., 2013). Further studies will be necessary to elucidate the possible effects of ketamine when administered at different ages and using different doses/times of injection as well as studies directed at examining the mechanisms of action.

In rodents, the CUS paradigms produces anhedonia, the loss of interest in normally pleasurable and rewarding activities, which are a core symptom of depression (Ma et al., 2013; Willner, Muscat, & Papp, 1992). As based upon the results of our sucrose preference test, the CUS paradigm used in this report induced anhedonia, as rats exposed to chronic stress consumed significantly lower amounts of sucrose (Figure 3a). However, in response to ketamine, CUS rats showed increases in preferences for sucrose, indicating that they were now more responsive to the rewarding effects of sucrose. Anhedonia might provide insights into the underlying neurobiology of depression and changes in depression following treatments with antidepressants. With the use of anhedonia, it will be possible to identify areas and mechanisms associated with the sensitization of brain reward pathways (Subhan, Deslandes, Pache, & Sewell, 2000), resulting from enhanced firing activity dopamine neurons within the ventral tegmental area (Sekine, Suzuki, Ramachandran, Blackburn, & Ashby, 2007) and dopamine neurotransmission in the striatum (Stein, 2008). Interestingly, antidepressants alleviate (Katz & Carroll, 1977) or have no effects (Matthews et al., 1996) on responding for rewarding brain stimulation. However, it is important to note that NMDA receptor antagonists, such as AP‐1 and MK801, increase the total amount of food consumed in rodents (Treece, Ritter, & Burns, 2000).

Under certain conditions, behavioral activity can be used as an index of anxiety (Kovacs & de Wied, 1978). Our results showed that exposure to CUS produced a decrease in rats’ exploratory activity, an effect which was altered by short‐term ketamine treatment. This increase in exploratory activity can be interpreted as a reduction in the degree of anxiety in these ketamine‐treated rats. The work suggested the behavior of up‐right was on behalf of the rat's ability for adapting a strange environment (Casu et al., 2005). In contrast to our findings, the work of Marin, Cruz, & Planeta, (2007), revealed that animals exposed to chronic restraint stress displayed higher exploratory behavior in the open field test. It is possible that the categories, intensities and number of exposures to the stressors were important factors in determining how chronic stress affected the exploratory activity in response to a novel environment.

Anxiety‐like behavior, locomotor activity and risk assessment were evaluated with use of the EPM (Myers & Greenwood‐Van Meerveld, 2010). CUS rats showed increased amounts of anxiety‐like behavior, which was characterized by spending less time in the open arms and decreased locomotor activity in the EPM. In contrast, ketamine‐treated rats showed behavioral responses indicative of antianxiety‐like behavior. Taking these results obtained from both the OF and EPM tests, it seems that ketamine might be operating as anantidepressant drug.

In the FS test, rats adopt an immobile posture after an initial period of vigorous activity of swimming in a beaker from which they cannot escape, and this immobility has been interpreted as reflecting despair/depression (Aan het Rot et al., 2010; Murrough et al., 2013). Ketamine‐treated rats demonstrated lower levels of despair when subjected to the FS test, as indicated by showing increased immobility latencies as compared with that of the CUS + Vehicle group following the long‐term treatment with ketamine. A related study failed to find any such antidepressant effects of ketamine (Hayase, Yamamoto, & Yamamoto, 2006). However, in that study, rats were forced to swim for an extended period of time, which may have reduced the influence of ketamine on the responses of these rats (Parise et al., 2013).

Collating the findings from the array of behavioral assays used in this report, we demonstrate that ketamine yielded rapid antidepressant‐like effects in rats exposed to CUS conditions, effects which were greater that observed with long‐term treatment. Parise et al. revealed that ketamine produced rapid antidepressant responses in rodents, however, these antidepressant or anxiolytic effects were not sustained (Parise et al., 2013) and findings from other studies have indicated that the antidepressant responses to ketamine only lasted for a few days (Autry et al., 2011; Maeng et al., 2008). There is also evidence from clinical reports that acute ketamine injection yielded rapid antidepressant and anxiolytic effects (Aan Het Rot, Zarate, Charney, & Mathew, 2012; Sappington, Corssen, Becker, & Tavakoli, 1977), however, these antidepressant and anxiolytic symptoms relapsed within days (Aan Het Rot et al., 2012; Kindler et al., 2013). While, a study by Murrough JW has reported that repeated ketamine administration was being explored as a treatment paradigm for the long‐lasting maintenance of antidepressant response (Aan het Rot et al., 2010; Murrough et al., 2013). For analysis of the long‐term effects of ketamine, the CUS + Ketamine group showed significantly increased activity as compared with the CUS + Vehicle group. Thus, our studies offered a new strategy consisting of a 14‐day ketamine administration that ameliorates CUS. The repeated ketamine administration might lead to robust, perhaps permanent, changes in cortical synapses for keeping longer lasting antidepressant effects (Parise et al., 2013). Meanwhile, we cannot discard the idea that repeated ketamine administration caused PV‐loss in the PFC (Yang, Han, Zhang, Ren, & Hashimoto, 2016), suggesting a detrimental side effects of repeated ketamine injections which might lead to the effectless of the antidepressant response to the long‐term treatments. And the potential metabolic side effects that may result from these treatments, especially on the dose and repeated injection time and frequency will require future research.

A single training session with use of the WM test was used to differentiate the drug's effects on different stages of memory. While the conventional protocols of WM test involved repetitive multitraining sessions in several days, such a protocol makes it difficult to differentiate the drug's effects on different memory aspects, as suggested by Moosavi, Yadollahi Khales, Rastegar, & Zarifkar, (2012). Results obtained from previous studies had indicated that ketamine impaired memory and learning behavior as the rats treated with ketamine spent more time to reach the hidden platform (Walker & Gold, 1991). Moreover, subanesthetic posttraining doses of ketamine (5 mg/kg) showed no effects on memory consolidation and larger doses (10, 20, and 50 mg/kg) did not influence the retrieval of memory when tested in a T‐maze (Wang, Fu, Wilson, & Ma, 2006). However, Getova and Doncheva reported that a subanesthetic administration of ketamine actually improved learning and memory as revealed in an active avoidance test (Getova & Doncheva, 2011). Our current results suggest that a subanesthetic dose of either short‐ or long‐term ketamine exerted no significant effects upon learning and working memory as assessed in the WM test (Figure 6). In fact, the escape latencies of the CUS + Ketamine group were somewhat longer than that obtained in the CUS + Vehicle group. Given that ketamine is a noncompetitive antagonist of the N‐methyl‐D‐aspartate (NMDA) receptor, which is considered to be one of the vitally important receptors involved with synaptic plasticity and neuronal learning (Rezvani, 2006), these findings are expected. It seems likely that these impairments in working memory by ketamine were not attributable to the dysfunction of motivational, motor, short‐term memory or spatial memory processes (Storer & Demeyere, 2014). While the water maze is generally considered to be a test of spatial learning and working memory in rodents (Brody & Holtzman, 2006), performance in this task could also be influenced by a number of other factors.

In summary, our study utilized a depression model in rats for use in evaluating the antidepressant effectiveness of short‐term and long‐term ketamine administration. We demonstrate that a 14‐day ketamine treatment not only induced a rapid onset, but also a persistent long‐term antidepressant effect in rats. The mechanisms underlying this effect, as well as any potential metabolic side effects that may result from these treatments, will require future research.

## 5 CONCLUSION

We conclude from this study that the use of a subanesthetic dose of ketamine to CUS exposed rats’ results in greater increases of weight gain, higher sensitivity to sucrose, augmented spontaneous locomotor behavior to anxiety‐eliciting situations, and some deficits in memory. Ketamine induced rapid antidepressant‐like effects in adult male rats exposed to CUS conditions; however, these antidepressant‐like effects were not present under conditions of long‐term ketamine treatment.

## CONFLICT OF INTEREST

None declared.
